# Systematic Identification of Candidate Genes for Inherited Retinal Disease Gene Therapy Integrating Worldwide IRD Cohort and Single-Cell Analysis

**DOI:** 10.1155/joph/7014745

**Published:** 2025-06-12

**Authors:** Ching-Yun Wang, Lawrence Chen, Ting-Yi Lin, Shun-Ping Huang

**Affiliations:** ^1^Department of Medical Education, Taichung Veterans General Hospital, Taichung, Taiwan; ^2^Department of Ophthalmology, Chang Gung Memorial Hospital, Chiayi, Taiwan; ^3^Biological Sciences Division, University of Chicago Pritzker School of Medicine, Chicago, Illinois, USA; ^4^Doctoral Degree Program of Translational Medicine, Academia Sinica, Taipei, Taiwan; ^5^Neurobiology, Neurodegeneration and Repair Laboratory, National Eye Institute, National Institutes of Health, Bethesda, Maryland, USA; ^6^Department of Ophthalmology, Taichung Tzu Chi Hospital, Taichung, Taiwan; ^7^Department of Biochemical Science and Technology, National Chiayi University, Chiayi, Taiwan

## Abstract

Inherited retinal dystrophies (IRDs) constitute a heterogeneous group of primarily monogenic orphan diseases caused by mutations in over 300 genes, collectively affecting millions of individuals worldwide with visual impairment. Despite significant advances, the development of gene replacement therapy for IRDs has predominantly focused on single-gene approaches, lacking a unified strategy driven by factors such as global prevalence, disease burden, and feasibility of therapeutic delivery. In this review, we propose a comprehensive protocol integrating multifaceted methodologies to refine the identification of suitable gene therapy candidates. We assessed gene prevalence, transcript size compatibility with vector packaging, and cell-type–specific expression, enabling the prioritization of promising therapeutic targets. Our approach focuses on assessing enzyme-coding genes as prime, more suitable candidates for therapeutic intervention due to their relatively similar replacement mechanism. Our findings provide a framework for identifying additional genes that may benefit from similar translational pipelines. This approach revealed a spectrum of potential candidates, including several underexplored genes with high therapeutic potential. Our findings underscore the necessity of adopting a strategic, data-driven framework to prioritize clinically impactful and scalable gene therapy targets, paving the way for broader and more effective therapeutic applications in the field of IRDs.

**Trial Registration:** ClinicalTrials.gov identifier: NCT01482195, NCT03316560, NCT06333249, NCT06275620, NCT04850118, NCT05926583

## 1. The Pearl and Unmet Needs in the Molecular Diagnosis Era of IRD

Inherited retinal dystrophies (IRDs) are a diverse group of rare disorders that progressively damage retinal photoreceptor cells, leading to irreversible blindness and affecting millions of individuals across various populations [1]. Although over 300 genes have been implicated in IRDs, developing effective gene therapies has been limited to a few well-characterized targets. The FDA approval of voretigene neparvovec (Luxturna) for retinal pigment epithelial (RPE) 65–associated retinal dystrophy has sparked hope for similar therapies targeting other IRD–causing genes [2, 3]. However, a systematic approach to identifying and prioritizing candidate genes for gene therapy has yet to be established. This study aims to fill this gap by integrating global IRD cohort data with single-cell expression analysis, focusing on enzyme-coding genes. These genes are particularly suitable for gene augmentation therapy due to their involvement in essential biochemical pathways, and their loss-of-function mutations often lead to severe phenotypes. By evaluating the prevalence of IRD–causing genes, their transcript length, and their expression in retinal cell types, we aim to provide a pivotal framework for selecting optimal gene therapy candidates.

Given the profound impact of IRDs on quality of life and the current lack of effective treatments, identifying viable gene therapy candidates has become a priority in ocular genetics and clinical research. Recent advancements in gene therapy offer promising strategies to correct the underlying genetic defects in IRDs. Approaches such as vector-based gene augmentation, antisense oligonucleotides, and cutting-edge genome editing techniques are actively being explored. Early trials have demonstrated the potential to restore vision in affected individuals. For example, voretigene neparvovec (Luxturna), the first FDA-approved gene therapy for an IRD, has significantly improved visual function in patients with RPE65 mutations following a one-time sub-retinal injection [4].

These advancements underscore the critical need to discover new gene therapy candidates targeting a broader range of genetic mutations associated with IRDs. While gene therapy holds significant promise, it is not without challenges. Concerns about long-term safety, immune responses, potential off-target effects, and delivery limitations of adeno-associated virus (AAV) vectors, particularly for large IRD genes, are still being investigated in clinical trials. By systematically outlining the current research landscape, this study aims to identify more effective therapeutic approaches that avoid some of these challenges.

With the advent of high-throughput panel sequencing, data on the molecular epidemiology of IRDs and the key genes contributing to IRDs in different countries are becoming available. In contrast to patient-level investigations, Hanany et al. [5] conducted a population-level analysis worldwide using the gnomAD database, examining carrier frequencies and the expected number of affected individuals for recessive IRD genes across diverse ancestries.

Although rare, disease market analysis is crucial for the development of gene therapy, population and IRD cohort analyses have not been integrated into the prioritization of candidate gene therapy development. This study aims to facilitate the integration of knowledge from existing literature. Moreover, we integrated gene targets with key gene therapy design considerations such as transcript size and cell-type specificity in expression using single-cell high-throughput data to characterize the attributes of the established IRD genes globally in an integrated cross-disciplinary approach.

## 2. Systemic Identification of Gene Supplement Therapy Candidates for IRDs

**Figure 1:**
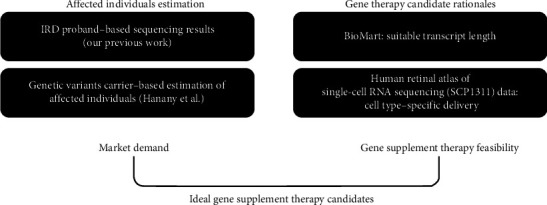
Flowchart of the integrated methodologies for identifying ideal AAV gene supplement therapy candidates.

To screen candidate genes for gene supplement therapy unbiasedly, we integrated multiple methodologies to achieve this goal, as demonstrated in this review. The screening protocols included an assessment of market demand and the feasibility of gene supplement therapy. In the first part, we utilized IRD proband–based sequencing results and estimated genetic variants carried by affected individuals. The second part involved querying the BioMart database for transcript length [6] and using the human retinal single-cell RNA sequencing (scRNA-seq) atlas. These combined methods collectively support the objective of identifying key IRD gene candidates and evaluating their potential for gene therapy (Figure 1).

## 3. IRD Proband–Based Sequencing: A Real-World Demonstration of the Frequently Captured Disease-Causing Genes

In our previous work [7], we depicted a global prevalence of disease-causing genes after pooling the molecular diagnostic results among IRD probands from 20 cross-continental studies (see Supporting Table 1). As a result, the top-10 leading disease-causing genes with the highest diagnostic yields were *ABCA4. CEP290, CRB1, CYPV42, EYS, GUCY2D, PDE6B, RHO, RPGR*, and *USH2A*. To reappraise the current understanding of these genes, we summarize the pathophysiology and gene therapy development status in Table 1.

## 4. Emerging Single-Cell Transcriptome Reshaped the Understanding of IRD Pathophysiology

**Figure 2:**
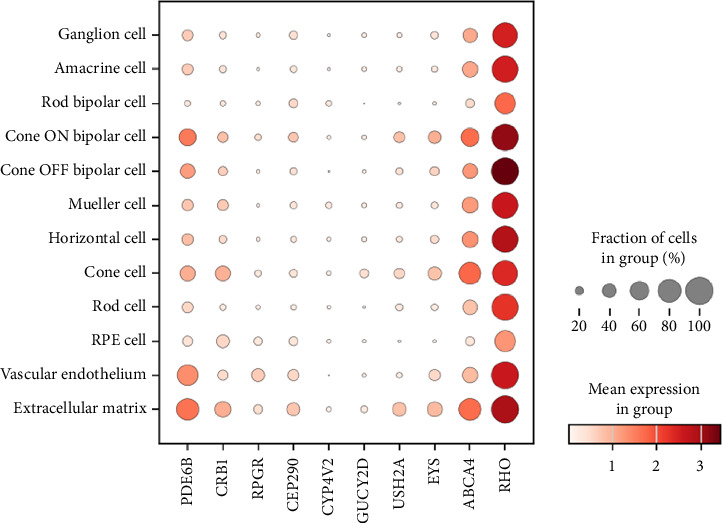
Cell-type–specific expressions of the top-10 genes contributing to IRD cases. Single-cell expression datasets are obtained from the study [41] via the single-cell portal.

To explore the cellular context of gene expression and its relevance to potential therapeutic targeting, we integrated the latest scRNA-seq data to investigate the expression of IRD–associated genes across different retinal cell types [41]. We analyzed and visualized the expressional profile of IRD genes at the single-cell level using the overlapped gene lists from *RetNet.org* and the open-source scRNA-seq data. To narrow the scope, we selectively presented the cell-type–specific expression of the top-10 genes with the highest diagnostic yields reported in the literature contributing to IRD cases (as described in Section 3): *ABCA4, CEP290, CRB1, CYPV42, EYS, GUCY2D, PDE6B, RHO, RPGR,* and *USH2A* (Figure 2). The cell-type–specific expression of all IRD genes is shown in Supporting Figure 1.

Among the top ten prevalent genes, no significant cell-type specific expression was observed across the gene expression profiles of the 12 retinal cell types investigated. *CYP4V2* and *GUCY2D* are ubiquitously expressed in lower levels, while *RHO* is abundantly expressed. The scRNA-seq atlas showed discordance with the previous knowledge of the pathogenic mechanism of some genes. For example, *ABCA4* mutation was correlated with impaired cone and rod photoreceptor conductivity, with some studies reporting delayed abolition of rod ERG long after the initial arCD/arCRD was diagnosed [42]. These inconsistent phenotypes might be partially explained by a relatively higher *ABCA4* expression level in cones than in rod photoreceptors (Figure 2). *CEP290* mutations are associated with photoreceptor disruption due to the impaired ciliogenesis of RPE [43] cells. However, *CEP290* was most abundant in the retinal extracellular matrix, which aligns with the emergence of ECM–directed retinogenesis [44, 45] and suggests a more complex pathogenesis linking *CEP290* variants to IRD phenotypes.

Recent advancements in gene therapy and molecular research have highlighted the importance of cell-type–specific gene expression profiles in minimizing off-target effects, reducing undesirable side effects, and optimizing treatment efficacy. However, our examination of cell-type–specific expression patterns revealed that many prevalent IRD genes exhibit a mostly ubiquitous expression across the diverse retinal cell types (see Figure 2, Supporting Figure 1). This finding contradicts the expectation of distinct expression profiles across different retinal cell types. Targeting specific cell types for gene therapy may overlook the broader, pan-tissue impacts of malfunctioning IRD genes and their interactions with various cell types in mediating disease. Focusing solely on individual cell types in therapeutic interventions may prove less effective, highlighting the need for a more comprehensive strategy that simultaneously targets multiple affected cell types.

These findings call for a paradigm shift in gene therapy approaches for IRDs. Rather than concentrating solely on single-cell targeting, a holistic strategy that concurrently addresses multiple cell types may provide a more effective therapeutic approach. Moreover, future IRD research should focus on refining current vector technologies to accommodate the genes currently under investigation and exploring alternative delivery systems to expand the scope of gene therapy options. Simultaneously, our results emphasize the necessity of a closer integration between research on IRD–causing genes and gene therapy development. When investigating disease-causing genes, it is crucial that researchers also consider their therapeutic feasibility.

IRD genes are expressed ubiquitously across various tissues; however, elucidating the specific causal cell types and the temporal dynamics mediating disease progression remains an area requiring further investigation. Advances in emerging technologies enable more precise exploration of this seemingly straightforward yet complex question within the intricate retinal environment. Recent studies highlight the critical need for genetic access to distinct retinal cell types to unravel their roles and trajectories throughout development and disease progression [46–48]. Notably, innovations in single-cell AAV engineering (scAAVengr) pipelines facilitate the systematic evaluation of viral vector efficiency across diverse retinal cell types [49]. Moreover, identifying pathogenic cell populations and targeting differentiated cells during specific developmental stages using genetic switches and synthetic promoters offer promising avenues for developing targeted therapies [50, 51].

scRNA-seq data from the adult retina provide valuable insights into cell-type–specific gene expression patterns. In principle, the epicenter of pathogenic events could be inferred based on mutations in specific IRD–associated genes. However, a significant challenge in establishing the cell-type–specificity of these genes lies in their temporal expression dynamics during retinal development. Caution is warranted when interpreting adult retinal scRNA-seq data, as such datasets lack longitudinal information about gene activation or deactivation during development and may not capture quantitative changes in gene expression over time. This limitation underscores the necessity for temporally resolved studies to enhance our understanding of retinal disease mechanisms.

## 5. Autosomal Recessive IRD (AR-IRD) Burden- and Cargo Size-Oriented Gene Therapy Candidate Selection

A comprehensive global market survey is essential for the successful development of orphan drugs, particularly in genetic therapeutics, where specific gene mutations further restrict patient eligibility. Beyond the mapped gene rankings derived from IRD probands undergoing molecular diagnosis, Hanany et al. [5] conducted an extensive analysis of variants across 187 IRD–associated genes, assessing the prevalence of pathogenic variant carriers within the general population. Their findings estimated approximately 2.7 billion carriers of IRD–causing variants globally, with 5.5 million affected individuals, including 3 million from the Asian population.

**Figure 3:**
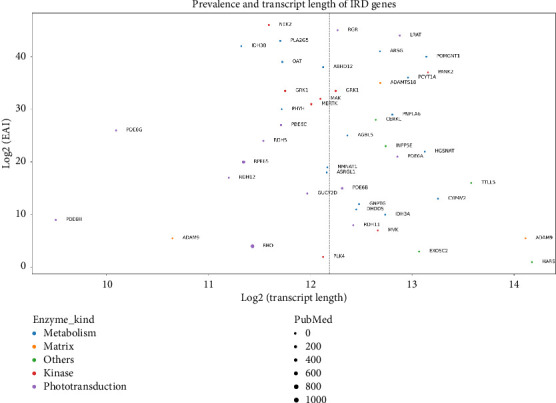
Expected affected individuals (EAI) vs transcript size scatterplot of IRD–causing genes. Transcript size is obtained from gene ensembles in the Gene Expression Omnibus (GEO) database using R (BioMart, GenomicFeatures package). EAI is obtained from the study by Hanany et al. via their SQL database [5]. Color: enzyme function. Bubble size: number of publications mentioning retina and the gene in the PubMed database. This scatterplot indicated that the contemporary research intensity on IRD genes is not proportionally aligned with their clinical relevance or potential therapeutic impact.

In addition to precise molecular epidemiological data, the development of gene supplementation therapies necessitates careful consideration of transcript length due to packaging constraints inherent in AAV vectors, which typically have a maximum capacity of approximately 4.7 kb. This technical limitation poses challenges for delivering large genes associated with certain IRDs. Conversely, enzyme-coding genes present an advantageous target for gene supplementation therapies due to their catalytic function, where even partial restoration of enzyme activity can result in significant therapeutic outcomes. For example, AADC gene therapy reestablishes dopamine synthesis in AADC deficiency [52], while 5%–20% enzyme restoration levels are often sufficient to correct metabolic imbalances, as demonstrated in therapies for sickle cell disease. The flexible intracellular localization of enzymes simplifies gene therapy design, enabling the correction of toxic metabolite accumulation or deficient product synthesis, such as converting levodopa to dopamine in Parkinson's disease. To evaluate the suitability of IRD–associated genes for AAV–mediated gene therapy, we compiled transcript length data from RetNet.org and cross-referenced these with BioMart. We then visualized the estimated affected individuals (EAIs) and corresponding transcript lengths of each IRD–associated gene (Figure 3), providing an integrated perspective on gene therapy feasibility within the constraints of AAV vector technology.

Several enzyme-coding genes, including *PDE6G, RDH5, PDE6C, PHYH, MERTK, MAK, GRK1, ABHD12, OAT, IDH3B, PLA2G5*, and *NEK2*, were identified as more prevalent IRD–causing genes than RPE65 and fell within the cargo size limit for AAV vectors (positioned to the left of the gray dashed line representing a transcript length of 4.7 kb). As of November 2024, only *MERTK* had progressed to clinical trials. Among these genes, *NEK2*, a serine/threonine–protein kinase–coding gene, exhibited the highest EAIs, suggesting a considerable unmet clinical need and market potential. Despite its promising profile, research into AAV–based gene therapy targeting *NEK2* remains limited. To further explore the translational potential of enzyme-coding IRD genes, we assessed their research prevalence in retina-related studies indexed in PubMed. While *RHO* and *RPE65* emerged as the most frequently investigated genes (Figure 3), many enzyme-coding genes with significant EAI remain underexplored. This stark contrast between the volume of published literature and the expected burden of disease indicates that research intensity on IRD genes is not necessarily aligned with their clinical relevance or potential therapeutic impact. We advocate for a more strategic approach to gene therapy research, prioritizing genes based on their functional significance, compatibility with AAV cargo size limitations, and the estimated population of loss-of-function variant carriers. Such a shift could accelerate the development of therapeutics with the most substantial translational potential, ultimately benefiting underserved patient populations.

Among the enzyme-coding genes identified as strong gene therapy candidates, *MERTK* stands out due to its well-established role in retinal homeostasis. *MERTK* encodes a receptor tyrosine kinase essential for phagocytosis of photoreceptor outer segments by RPE cells. Loss-of-function mutations in *MERTK* lead to a severe, early-onset form of retinitis pigmentosa (RP38), characterized by progressive photoreceptor degeneration [53–55]. The feasibility of gene augmentation therapy for *MERTK*-associated RP has been demonstrated in preclinical models, where AAV–mediated *MERTK* delivery restored photoreceptor survival and function. These findings emphasize the translational potential of enzyme-coding genes for gene therapy and further support their prioritization in IRD treatment strategies.

The transition from single-cell–type gene therapy to multicell-type targeting presents both opportunities and challenges. Current AAV serotypes, such as AAV2, AAV5, and AAV8, exhibit varying tropisms for photoreceptor RPE, and Müller glia [56]. However, natural AAV serotypes may not efficiently transduce all relevant retinal cell types simultaneously.

To overcome these limitations, engineered virus-like particles (eVLPs) have been developed to improve cargo packaging, release, and localization. Capsid mutations optimize the packaging and delivery of desired ribonucleoprotein cargos, rather than native viral genomes, and substantially alter eVLP's capsid structure [57]. In parallel, directed evolution strategies have been employed to generate novel AAV capsids with enhanced multicell-targeting capabilities [58]. For instance, AAV9-PHP.B has demonstrated superior transduction efficiency across multiple retinal layers.

Beyond capsid engineering, hybrid delivery systems, including dual AAV vectors and nanoparticle-based approaches, are being explored to overcome AAV's packaging constraints, particularly for large IRD genes. Another emerging approach is scAAVengr, which allows for the systematic evaluation of AAV variants at single-cell resolution [59]. This approach enables the identification of AAV capsids with optimal transduction profiles for specific retinal cell types, improving precision in gene therapy applications.

## 6. Conclusions

Our study contributes to the growing knowledge of gene therapy for IRDs. Building on the foundational work of Hanany et al., we addressed the predominantly ubiquitous expression of prevalent IRD–associated genes across retinal cell types. Advances in cell-targeting technologies considering multiple gene markers simultaneously could facilitate more precise therapeutic targeting. We identified potential enzyme-coding gene candidates for IRD gene therapy by evaluating their suitability for AAV–mediated delivery based on transcript length and estimated disease incidence. Our literature survey revealed that many of these genes remain underrepresented in current research efforts, suggesting a misalignment between research priorities and therapeutic potential. The current research landscape appears to prioritize genes with diagnostic value, which may have limited direct applicability for therapeutic development. Given the well-recognized suitability of enzyme-coding genes for traditional gene augmentation therapy due to their catalytic function and low expression threshold, our findings highlight their potential as promising targets for future IRD gene therapy initiatives. To maximize translational impact, future studies should prioritize underexplored enzyme-coding genes with favorable AAV–delivery profiles for preclinical validation, particularly those with high therapeutic potential but limited current representation in the IRD research landscape. This targeted focus could bridge the gap between gene identification and therapeutic application, ultimately accelerating progress toward effective gene therapies for IRDs.

## 7. Materials and Methods

### 7.1. Characteristics of IRD Genes

IRD–associated genes were collected from RetNet (https://retnet.org/) and mapped to the BioMart database to retrieve transcript length information. To estimate the global prevalence of IRD–associated variants, we used the dataset provided by Hanany et al., which reports the globally expected number of affected individuals based on allele frequencies from the gnomAD database [5].

In brief, to determine the EAIs for IRDs, Hanany et al. integrated population-level carrier frequencies with established models of autosomal recessive disease prevalence. Their approach was based on allele frequency data from the gnomAD database, demographic population statistics, and principles of carrier frequency (CF) and genetic prevalence (GP) calculations.

To estimate EAI, Hanany et al., applied a product-based algorithm to allele matrices. For each gene, we computed the probability of two heterozygous carriers transmitting an AR-IRD–causing mutation to their offspring. The total EAI was derived by summing the GP of all possible homozygous and compound heterozygous combinations. Demographic data were sourced from the United Nations 2017 World Population Prospects, ensuring that EAI estimates reflected global population distributions.

### 7.2. Global IRD Gene Prevalence

To enhance the reliability of EAI calculations, we applied gene-level filtering using real-world IRD cohorts. We prioritized the top-10 IRD genes based on their diagnostic yield in patient cohorts, ensuring that EAI estimates align with actual disease prevalence rather than relying solely on theoretical Mendelian assumptions from population data. According to our previous work [7], we filtered PubMed-acquired studies related to panel sequencing and IRDs (see Supporting Figure 1). We collected the gene prevalence data and ranked the top contributing genes via the percentage sum of IRD patients receiving the genetic molecular diagnosis. The publication information on the literature used is presented in Supporting Table 1.

To determine the global prevalence of IRD–associated genes, we conducted a systematic literature review using PubMed-acquired studies related to panel sequencing and IRDs (see Supporting Figure 1). We applied the following inclusion criteria: (1) studies using next-generation sequencing (NGS) panels or whole-exome sequencing (WES) for IRD diagnosis, (2) studies reporting genetic molecular diagnosis rates per gene, and (3) studies with sample sizes greater than 50 individuals to ensure statistical reliability. Publications that did not meet these criteria were excluded to minimize bias.

We extracted gene prevalence data from each selected study and standardized diagnostic rates by calculating the percentage of IRD patients receiving a molecular diagnosis for each gene. To minimize variability due to differences in sequencing platforms, cohort compositions, and geographic regions, we applied meta-analytical approaches to combine prevalence estimates. The final ranked list of top contributing IRD genes was generated using a weighted averaging method, prioritizing studies with larger cohort sizes and higher sequencing depth.

To validate our findings, we cross-referenced our ranked gene list with external IRD prevalence databases and population genetic datasets (e.g., gnomAD and ClinVar). In addition, we performed sensitivity analyses, evaluating the impact of excluding studies with small sample sizes or nonuniform diagnostic criteria. The final publication information on the literature used is provided in Supporting Table 1.

By integrating genetic carrier frequencies, demographic modeling, and real-world IRD cohort data, we generated a more clinically relevant estimate of affected individuals, improving the accuracy of disease burden predictions for IRD gene therapy prioritization.

### 7.3. Bioinformatics Analysis

The scRNA-seq data for the human retina were obtained from the Gene Expression Omnibus (GEO) under the accession code “GSE147979 [3].” To ensure transparency and reproducibility, we processed these data using a standard bioinformatics pipeline within the Jupyter notebook environment [60]. After downloading the scRNA-seq count matrix and converting it into an AnnData object, we applied the bioconductor workflow for scRNA-seq analysis, ensuring a robust preprocessing of the data [61].

After acquiring the AnnData format of scRNA-seq data, we chiefly used the Python toolkit “scanpy Version 1.9.5” for the downstream analyses [62]. The inherent variation caused by mitochondrial gene expression was regressed by the “scanpy.pp.regress_out” function. To identify cell clusters, principal component analysis (PCA) was first performed on the list of genes with higher variation than expected (highly variable genes). The highly variable genes were extracted by the “scanpy.pp.highly_variable_genes” function and selected with the following thresholds: “min_mean = 0.0125, max_mean = 3, min_disp = 0.5.” The numbers of significant PCs used in each PCA were determined by the Jackstraw method and are as follows: 31 PCs for human retina. Cell clusters were generated by an improved version of the Louvain algorithm, the “scanpy.tl.leiden” function [63]. To validate cluster identities, major cell classes were manually annotated based on the cluster-specific markers. We used a previous study by Tedja et al. to identify cluster biomarkers as a reference [64]. To confirm the expression specificity of IRD–associated genes, we conducted cross-validation using previously published bulk RNA-seq and spatial transcriptomic datasets, ensuring that observed scRNA-seq expression patterns were consistent with literature-reported cell-type expression. In addition, we compared our gene expression data with single-cell atlases of the human retina, verifying that known IRD genes exhibited expected cell-type enrichment patterns.

Finally, we generated a dot plot using the “scanpy.pl.dotplot” function to visualize the expression of the top-10 IRD genes across retinal cell types, providing critical insights into the cell-specific expression of genes relevant for potential gene therapy.

## Figures and Tables

**Table 1:** Characteristics of the 10 IRD disease–causing genes with relatively high diagnostic yields in IRD probands worldwide.

Gene name	Disease	Prevalence	Affected cell types	Pathophysiology	Gene therapy under clinical trials (until 31^st^ Nov, 2024)	References
ABCA4	*Stargardt* disease	1:8000–1:10,000	Photoreceptors	Impaired transmembrane transportation and toxic metabolites' accumulation in RPE	NCT06467344 NCT06445322 NCT06300476 NCT05956626	[8, 9]

CEP290	*Leber*'s congenital amaurosis (LCA)	30% of LCA	Photoreceptors	Impaired ciliogenesis and ciliary trafficking, leading to disorganized outer photoreceptor segments (cilia)	NCT05203939	[10–12]

CRB1	LCA8	10% of LCA; 2.7% of autosomal recessive RP (ArRP)	Apical RPE junction	Outer limited membrane defect	N/A	[13–15]

CYPV42	Bietti crystalline dystrophy (BCD)	0.5% in China	RPE	Thinning and loss of the RPE layer, interruption and loss of the interdigitation zone (IZ) and ellipsoid zone (EZ).	N/A	[16]

EYS	Retina pigmentosa (RP)	5%–30% ArRP	Photoreceptors	Disrupted photoreceptor structure, and crippled protein trafficking to outer segments (Oss).	N/A	[17, 18]

GUCY2D	ArLCA,; Autosomal dominant cone-rod dystrophy (AdCRD)	10%–20% of ArLCA	Photoreceptors	Impaired intracellular cGMP synthesis	NCT03920007	[19, 20]

PDE6B	ArRP	Not specified	Photoreceptors	Impaired intracellular cGMP hydrolysis resulted in photoreceptor toxicity.	NCT03328130	[21, 22]

RHO	Autosomal dominant RP (AdRP)	20%–30% of AdRP cases	Rod photoreceptor	Misfolded rhodopsin leads to photoreceptor cell death	NCT06388200 NCT05805007 NCT05203939	[23–27]

RPGR	X-linked RP	70%–90% of X-linked RP (XLRP)	Photoreceptors	Impaired protein transport within photoreceptors	NCT03252847 NCT03316560 NCT06333249 NCT06492850 NCT06275620 NCT04312672 NCT04517149 NCT04794101 NCT04671433 NCT04850118 NCT05874310 NCT03584165 NCT03116113 NCT05926583	[28–34]

USH2A	Usher syndrome Type II	57%–79% of Usher syndrome Type II	Photoreceptors and cochlear hair cells	Defective usherin protein affects photoreceptor and hair cell function	N/A	[35–40]

*Note:* The gene list was acquired from our previous work [6] (Supporting Figure 1). This table indicated a substantial gap between having high molecular diagnostic yields and being a suitable candidate for gene supplement therapy.

Abbreviation: N/A, not available.

## Data Availability

All data used to support the findings of this study are available from the corresponding author upon request.

## Supporting Information

Supporting Figure 1. Complete gene expression chart for each specific retina cell type along with the top-10 most prevalent genes.

Supporting Table 1. Publication information on the literature referenced.
