# Supplementary material for: Systematic Identification of Candidate Genes for Inherited Retinal Disease Gene Therapy Integrating Worldwide IRD Cohort and Single-Cell Analysis
Source: J Ophthalmol. 2025 Jun 12;2025:7014745. doi: 10.1155/joph/7014745 (PMC12178778; doi:10.1155/joph/7014745)
Supplement: Supporting Information — Additional supporting information can be found online in the Supporting Information section. [file 7014745.f1.docx]

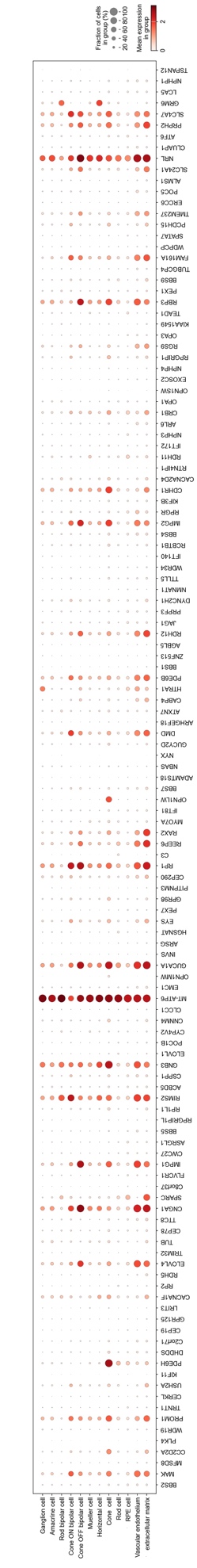


**Supplemental Figure 1.** Complete gene expression chart. Complete gene expression chart for each specific retina cell type along with the top 10 most prevalent genes

| **Author** | **Year** | **Country** | **Ancestry** | **Dx rate (%)** | **Ref** |
| --- | --- | --- | --- | --- | --- |
| Arai | 2015 | Japan | East asia | 45.6 | ^1^ |
| Bernardis | 2016 | Italy | European | 59.1 | ^2^ |
| Bohorquez | 2021 | Spanish | Latin America | 57.6 | ^3^ |
| Chen | 2021 | Taiwan (TIP) | East asia | 57.1 | ^4^ |
| Eisenberger | 2013 | Germany | European | 70 | ^5^ |
| Ellingford | 2016, 2018 | UK | European | 51, 79 | ^6^ |
| Ge | 2015 | USA, Canada | European | 49.5 | ^7^ |
| Haer-Wigman | 2017 | Dutch | European | 49 | ^8^ |
| Holtan | 2020 | Norway | European | 32 | ^9^ |
| Huang(a) | 2015, 2018 | Chinese | East asia | 55.3, 64.6 | ^10,11^ |
| Jespersgaard | 2019 | Denmark | European | 71.9 | ^12^ |
| Kao | 2023 | Taiwan | East asia | 68.568.5%) | ^13^ |
| Kim | 2019 | Korea | East asia | 44.2 | ^14^ |
| Liu | 2020, 2021 | China | East asia | 44.5, 57 | ^15^ |
| Motta | 2019 | Brazil | Latin America | 76.8 | ^16^ |
| Perea-Romero | 2021 | Spain | Latin America | 53.2 | ^17^ |
| Pontikos | 2020 | French | European | 71.8 | ^18^ |
| Sheck | 2021 | UK | European | 59.4 | ^19^ |
| Stone | 2017 | USA | European | 76 | ^20^ |
| Wang | 2015, 2018 | China | East asia | 76.6, 41.2 | ^21^ |
| Weisschuh | 2020 | Germany | European | 70.8 | ^22^ |

**Supplementary Table 1**. Publication information on the literature referenced.

**Reference:**

1. Arai, Y., Maeda, A., Hirami, Y., Ishigami, C., Kosugi, S., Mandai, M., Kurimoto, Y., and Takahashi, M. (2015). Retinitis Pigmentosa with EYS Mutations Is the Most Prevalent Inherited Retinal Dystrophy in Japanese Populations. J Ophthalmol *2015*, 819760. https://doi.org/10.1155/2015/819760.

2. Bernardis, I., Chiesi, L., Tenedini, E., Artuso, L., Percesepe, A., Artusi, V., Simone, M.L., Manfredini, R., Camparini, M., Rinaldi, C., et al. (2016). Unravelling the Complexity of Inherited Retinal Dystrophies Molecular Testing: Added Value of Targeted Next-Generation Sequencing. Biomed Res Int *2016*, 6341870. https://doi.org/10.1155/2016/6341870.

3. García Bohórquez, B., Aller, E., Rodríguez Muñoz, A., Jaijo, T., García García, G., and Millán, J.M. (2021). Updating the Genetic Landscape of Inherited Retinal Dystrophies. Front Cell Dev Biol *9*, 645600. https://doi.org/10.3389/fcell.2021.645600.

4. Chen, T.-C., Huang, D.-S., Lin, C.-W., Yang, C.-H., Yang, C.-M., Wang, V.Y., Lin, J.-W., Luo, A.C., Hu, F.-R., and Chen, P.-L. (2021). Genetic characteristics and epidemiology of inherited retinal degeneration in Taiwan. NPJ Genom Med *6*, 16. https://doi.org/10.1038/s41525-021-00180-1.

5. Eisenberger, T., Neuhaus, C., Khan, A.O., Decker, C., Preising, M.N., Friedburg, C., Bieg, A., Gliem, M., Charbel Issa, P., Holz, F.G., et al. (2013). Increasing the yield in targeted next-generation sequencing by implicating CNV analysis, non-coding exons and the overall variant load: the example of retinal dystrophies. PLoS One *8*, e78496. https://doi.org/10.1371/journal.pone.0078496.

6. Ellingford, J.M., Barton, S., Bhaskar, S., O’Sullivan, J., Williams, S.G., Lamb, J.A., Panda, B., Sergouniotis, P.I., Gillespie, R.L., Daiger, S.P., et al. (2016). Molecular findings from 537 individuals with inherited retinal disease. J Med Genet *53*, 761–767. https://doi.org/10.1136/jmedgenet-2016-103837.

7. Ge, Z., Bowles, K., Goetz, K., Scholl, H.P.N., Wang, F., Wang, X., Xu, S., Wang, K., Wang, H., and Chen, R. (2015). NGS-based Molecular diagnosis of 105 eyeGENE(®) probands with Retinitis Pigmentosa. Sci Rep *5*, 18287. https://doi.org/10.1038/srep18287.

8. Haer-Wigman, L., van Zelst-Stams, W.A., Pfundt, R., van den Born, L.I., Klaver, C.C., Verheij, J.B., Hoyng, C.B., Breuning, M.H., Boon, C.J., Kievit, A.J., et al. (2017). Diagnostic exome sequencing in 266 Dutch patients with visual impairment. Eur J Hum Genet *25*, 591–599. https://doi.org/10.1038/ejhg.2017.9.

9. Holtan, J.P., Selmer, K.K., Heimdal, K.R., and Bragadóttir, R. (2020). Inherited retinal disease in Norway - a characterization of current clinical and genetic knowledge. Acta Ophthalmol *98*, 286–295. https://doi.org/10.1111/aos.14218.

10. Huang, H., Chen, Y., Chen, H., Ma, Y., Chiang, P.-W., Zhong, J., Liu, X., Asan, null, Wu, J., Su, Y., et al. (2018). Systematic evaluation of a targeted gene capture sequencing panel for molecular diagnosis of retinitis pigmentosa. PLoS One *13*, e0185237. https://doi.org/10.1371/journal.pone.0185237.

11. Huang, X.-F., Huang, F., Wu, K.-C., Wu, J., Chen, J., Pang, C.-P., Lu, F., Qu, J., and Jin, Z.-B. (2015). Genotype-phenotype correlation and mutation spectrum in a large cohort of patients with inherited retinal dystrophy revealed by next-generation sequencing. Genet Med *17*, 271–278. https://doi.org/10.1038/gim.2014.138.

12. Jespersgaard, C., Fang, M., Bertelsen, M., Dang, X., Jensen, H., Chen, Y., Bech, N., Dai, L., Rosenberg, T., Zhang, J., et al. (2019). Molecular genetic analysis using targeted NGS analysis of 677 individuals with retinal dystrophy. Sci Rep *9*, 1219. https://doi.org/10.1038/s41598-018-38007-2.

13. Kao, H.-J., Lin, T.-Y., Hsieh, F.-J., Chien, J.-Y., Yeh, E.-C., Lin, W.-J., Chen, Y.-H., Ding, K.-H., Yang, Y., Chi, S.-C., et al. (2024). Highly efficient capture approach for the identification of diverse inherited retinal disorders. NPJ Genom Med *9*, 4. https://doi.org/10.1038/s41525-023-00388-3.

14. Kim, M.S., Joo, K., Seong, M.W., Kim, M.J., Park, K.H., Park, S.S., and Woo, S.J. (2019). Genetic Mutation Profiles in Korean Patients with Inherited Retinal Diseases. J Korean Med Sci *34*, e161. https://doi.org/10.3346/jkms.2019.34.e161.

15. Liu, X., Tao, T., Zhao, L., Li, G., and Yang, L. (2021). Molecular diagnosis based on comprehensive genetic testing in 800 Chinese families with non-syndromic inherited retinal dystrophies. Clin Exp Ophthalmol *49*, 46–59. https://doi.org/10.1111/ceo.13875.

16. Motta, F.L., Martin, R.P., Filippelli-Silva, R., Salles, M.V., and Sallum, J.M.F. (2018). Relative frequency of inherited retinal dystrophies in Brazil. Sci Rep *8*, 15939. https://doi.org/10.1038/s41598-018-34380-0.

17. Perea-Romero, I., Gordo, G., Iancu, I.F., Del Pozo-Valero, M., Almoguera, B., Blanco-Kelly, F., Carreño, E., Jimenez-Rolando, B., Lopez-Rodriguez, R., Lorda-Sanchez, I., et al. (2021). Genetic landscape of 6089 inherited retinal dystrophies affected cases in Spain and their therapeutic and extended epidemiological implications. Sci Rep *11*, 1526. https://doi.org/10.1038/s41598-021-81093-y.

18. Pontikos, N., Arno, G., Jurkute, N., Schiff, E., Ba-Abbad, R., Malka, S., Gimenez, A., Georgiou, M., Wright, G., Armengol, M., et al. (2020). Genetic Basis of Inherited Retinal Disease in a Molecularly Characterized Cohort of More Than 3000 Families from the United Kingdom. Ophthalmology *127*, 1384–1394. https://doi.org/10.1016/j.ophtha.2020.04.008.

19. Sheck, L.H.N., Esposti, S.D., Mahroo, O.A., Arno, G., Pontikos, N., Wright, G., Webster, A.R., Khan, K.N., and Michaelides, M. (2021). Panel-based genetic testing for inherited retinal disease screening 176 genes. Mol Genet Genomic Med *9*, e1663. https://doi.org/10.1002/mgg3.1663.

20. Stone, E.M., Andorf, J.L., Whitmore, S.S., DeLuca, A.P., Giacalone, J.C., Streb, L.M., Braun, T.A., Mullins, R.F., Scheetz, T.E., Sheffield, V.C., et al. (2017). Clinically Focused Molecular Investigation of 1000 Consecutive Families with Inherited Retinal Disease. Ophthalmology *124*, 1314–1331. https://doi.org/10.1016/j.ophtha.2017.04.008.

21. Wang, L., Zhang, J., Chen, N., Wang, L., Zhang, F., Ma, Z., Li, G., and Yang, L. (2018). Application of Whole Exome and Targeted Panel Sequencing in the Clinical Molecular Diagnosis of 319 Chinese Families with Inherited Retinal Dystrophy and Comparison Study. Genes (Basel) *9*, 360. https://doi.org/10.3390/genes9070360.

22. Weisschuh, N., Obermaier, C.D., Battke, F., Bernd, A., Kuehlewein, L., Nasser, F., Zobor, D., Zrenner, E., Weber, E., Wissinger, B., et al. (2020). Genetic architecture of inherited retinal degeneration in Germany: A large cohort study from a single diagnostic center over a 9-year period. Hum Mutat *41*, 1514–1527. https://doi.org/10.1002/humu.24064.
